# New Natural and Sustainable Cosmetic Preservative Based on Sugarcane Straw Extract

**DOI:** 10.3390/molecules29163928

**Published:** 2024-08-20

**Authors:** Maria João Carvalho, Sílvia Santos Pedrosa, Manuela Pintado, Ana L. S. Oliveira, Ana Raquel Madureira

**Affiliations:** CBQF—Centro de Biotecnologia e Química Fina—Laboratório Associado, Escola Superior de Biotecnologia, Universidade Católica Portuguesa, Rua Diogo Botelho 1327, 4169-005 Porto, Portugal; mjcarvalho@ucp.pt (M.J.C.); sspedrosa@ucp.pt (S.S.P.); mpintado@ucp.pt (M.P.); rmadureira@ucp.pt (A.R.M.)

**Keywords:** cosmetics, preservative, sugarcane, sustainable ingredients

## Abstract

Preservative ingredients in cosmetic formulations undertake a necessary role in the prevention of microbial contamination. In this field, there is an unmet need for natural, sustainable, and effective preservatives. Thus, the main goal of this work was to evaluate a sugarcane straw extract-based ingredient and investigate its potential as a preservative for cosmetic applications. Different ingredients were developed using several cosmetic solvents to improve the solubility of the extracted compounds. The antimicrobial activity was assessed against *Staphylococcus aureus*, *Escherichia coli*, *Pseudomonas aeruginosa*, and *Candida albicans*. The 1,2-hexanediol was the solvent that allowed us to achieve the ingredient (20% dry extract dispersed in 25% 1,2-hexanediol in water) with the best antimicrobial performance, showing a minimum inhibitory concentration of between 5% and 3% (*I*). The 5% (*w*/*v*) concentration of this ingredient complied with the USP51 standards for cosmetic preservatives. Real-time (25 °C, 65% RH) and accelerated stability (40 °C, 75% RH) tests were conducted to determine the ingredient stability, and it was found that one month of storage time at room temperature would be ideal for better ingredient stability and performance in terms of composition, pH, color, and antioxidant activity.

## 1. Introduction

Cosmetic formulations contain high amounts of water and nutrients, which provide an ideal environment for microorganisms to grow and proliferate. Microbial contamination represents a risk for cosmetic products as it can lead to product degradation and may negatively impact skin health, leading to infection [1]. To prevent microbial contamination, preservatives are added to cosmetic formulations to control microbial content during storage and usage [2]. Parabens are among the most well-known chemical preservatives used in the cosmetic industry. However, their toxicity has been linked to these compounds and their safety is always being questioned. Consequently, there are now more constraints from international legislation and directives as they advise using substances that are recognized as safe [3]. Therefore, the cosmetic industry is looking for alternatives to these chemical preservatives and is focusing on natural preservatives, such as phenolic-enriched extracts [4]. Several natural extracts have been reported to have preservative activity, including grapefruit seed [5], *Santolina chamaecyparissus* [6], and *Rubus rosaefolius* extracts [7]. Furthermore, currently in the market, there is a commercial cosmetic preservative composed of natural extracts, Microcurb™ OC, a mixture of caprylic acid and *Origanum vulgare* leaf extract.

A sugarcane straw is a byproduct of sugar and ethanol production. This material is known to be enriched in several phenolic compounds and organic acids, with several biological properties. Therefore, strategies to extract these biologically active compounds have been developed, and their activities have been characterized [8]. Sugarcane straw phenolic-enriched extracts have been shown to exhibit antimicrobial activity [9]. Through a variety of ways, phenolic compounds can inhibit or kill microbes. The degradation of the cell wall, damage to the membrane proteins and cytoplasmic membrane, leakage of the contents of the cell, and cytoplasm coagulation are a few of these [10]. The number and placement of the hydroxyl groups determine the antibacterial properties of certain phenolic compounds. Enhanced hydroxylation causes greater inhibition by oxidized phenols and enhanced toxicity against certain bacteria [11,12].

This work aims to investigate a natural and sustainable sugarcane straw extract-based cosmetic ingredient and study its antimicrobial activity against the most important cosmetic contaminant microorganisms. Further, the best performing ingredient will be evaluated through the USP 51 challenge test for preservative efficacy. Finally, the ingredient formulation stability will be tested in real-time and accelerated stability conditions. 

## 2. Results and Discussion

### 2.1. Ingredient Formulation

The sugarcane straw extract’s solubility in various cosmetic solvents was studied to develop a cosmetic ingredient to be used and incorporated in cosmetic formulations. Sugarcane straw extract is composed of hydroxybenzoic acids, hydroxycinnamic acids, and flavones [8]. 

Recent studies have underscored the significant antimicrobial efficacy of hydroxybenzoic acids, hydroxycinnamic acids, and flavones. Derivatives of hydroxybenzoic acids, such as gallic acid, have demonstrated potent antimicrobial properties against various bacteria, including *E. coli* and *S. aureus*, by disrupting bacterial cell membranes and leading to cell death [13]. Similarly, hydroxycinnamic acids like caffeic acid have shown synergistic effects when used in combination with antibiotics, particularly against antibiotic-resistant strains of *E. coli*, highlighting their potential in combating drug-resistant infections [14]. Furthermore, flavones, including those found in ethyl-4-ethoxybenzoic acid, have been identified as effective inhibitors of biofilm formation in *S. aureus*. These compounds alter cell membrane properties, enhancing the sensitivity of bacterial biofilms to antibiotics such as vancomycin [15].

The solubility of these compounds is often affected by their structural differences, namely molecular weight, that affects their polarity level, conjugation, and interaction with different matrixes [16]. Also, the extraction conditions comprehended the use of an ethanolic solution (50% (*v*/*v*)), which originated an extract that is not fully soluble in organic or aqueous solvents but in a mixture of both. Plus, ethanolic solutions at higher concentrations are not recommended solvents for cosmetic applications, since they can destroy the skin sebum layer, making it dry and cracked [17]. Therefore, there was a need to develop a soluble ingredient mixture using solvents usually employed in cosmetic formulations. Also, these solvents can improve the ingredient stability in a formulation, uniform extract dispersion within the formulation while exerting a booster effect.

Testing the solubility of the extract at different concentrations, with different solvents, and at different solvent to water proportions was performed as seen in Table 1. The purpose of this procedure was to have the maximum amount of extract powder dispersed in a solvent to obtain a more available bioactive ingredient. Among the solvents tested, only mixtures of 1,2-pentanediol, 1,2-hexanediol, dipropylene glycol, and 1,5-pentanediol with water at the proportions indicated in Table 1 were revealed to be effective in solubilizing the extract. The maximum amount of extract powder dispersed in the solvents mixtures was 20% for all of them except dipropylene glycol, which was able to solubilize 30% (*w*/*v*) of dry extract. The four ingredients resulting from the solubilization of the extract with 1,2-pentanediol, 1,2-hexanediol, dipropylene glycol, and 1,5-pentanediol (Table 1) were further studied regarding their performance. These solvents have antimicrobial activity and lower toxicity, therefore some of them have been used as co-preservatives, replacing traditional preservatives such as parabens, potassium sorbate, and sodium benzoate [18]. Even though they display activities of their own, it is believed that conjugation with antimicrobial compounds can boost their effect [19]. Furthermore, they also act as moisturizers, emulsion stabilizers, solvents for polar substances, dispersers of polymers, and as solubilizers [20]. 

### 2.2. Antimicrobial Activity

#### 2.2.1. Sugarcane Straw Extract

The sugarcane straw extract’s minimum inhibitory concentration (MIC_80_) and minimum bactericidal concentration (MBC) were determined by first dissolving the extract in water, where the insoluble part of the extract was then lost during the filtration step. The extract was evaluated against common contaminants of cosmetic products, including *S. aureus*, *E. coli*, *P. aeruginosa* and *C. albicans*. These microorganisms’ strains are known cosmetic contaminants [2]. 

Table 2 reflects the specific concentrations tested for each solvent, including the 30% extract concentration for dipropylene glycol as indicated in Table 1. This was performed to ensure consistency in evaluating the solubility and antimicrobial efficacy across different solvents. As seen in Table 2, the extract exhibits an MIC of 5% (*w*/*v*) against *S. aureus* and *E. coli*. Since phenolic compounds and organic acids have been documented to have an antimicrobial action [21], their presence in the extract can justify its antibacterial activity. Previous studies have highlighted the antimicrobial properties of the phenolic compounds extracted from sugarcane byproducts. A similar result was obtained when the same extract was tested against food-borne pathogens, in which the extract also displayed an MIC of 5% (*w*/*v*) against *S. aureus* [9]. A phenolic extract from sugarcane bagasse showed antimicrobial activity against *S. aureus* and *E. coli*, with an effective concentration of 0.06 and 0.25% (*w*/*v*), respectively [12]. These results are in accordance with the ones reported in this work, where a stronger activity against *S. aureus* was visible.

The soluble phenolic compounds are predicted to exert their antimicrobial activity at the membrane level [22]. *S. aureus* is more sensitive to these compounds due to the absence of an outer membrane, characteristic of Gram-positive bacterium, which facilitates their diffusion [12]. Likewise, the *E. coli* membrane allows small phenolic compounds such as phenolic acids, to cross the membrane and exert their antimicrobial activity [23]. Conversely, the membrane of *P. aeruginosa* contains a transport system that pumps toxins out of the cell, making it more resistant to the action of phenolic compounds. *C. albicans* is also more resistant to the action of phenolic compounds, since it contains a cell wall consisting of glucans, chitin, and proteins that block their action [23]. 

These results show that the sugarcane straw extract is capable of inhibiting *S. aureus* and *E. coli*. Given this antimicrobial capacity, the extract has been further formulated as a cosmetic ingredient to verify its efficacy once mixed in the cosmetic solvent.

#### 2.2.2. Sugarcane Straw Extract-Based Ingredients

After determining the antimicrobial activity of the extract, the four ingredients formulated and the respective solvent controls were tested at 5, 4, 3, 2, 1, and 0.5% (*w*/*v*) for their antimicrobial capacity through the evaluation of MIC and MBC. 

The ingredients obtained with the extract and dipropylene glycol and the extract with 1,5-pentanediol did not display antimicrobial activity against the microorganisms tested, and this is in accordance with the literature, where no MIC values or antimicrobial activity have been reported for this solvent [24,25]. Furthermore, 1,5-Pentanediol has been described to have antimicrobial activity against *S. aureus*, *E. coli*, and *P. aeruginosa* at concentrations of between 5% and 12% (*v*/*v*) [24]. Another study reported MIC values of 5% and 9% (*v*/*v*) against *C. albicans* and *S. aureus*, respectively [25]. These reports indicate that only at higher concentrations (≥5%) of direct solutions, 1,5-pentanediol has an antimicrobial effect, which explains the absence of antimicrobial activity in this study. The remaining two ingredients, the extract with 1,2-pentanediol and the extract with 1,2-hexanediol, both demonstrated antimicrobial activity. As seen in Table 2, the first only showed activity against Gram-negative bacteria, whereas the second exhibited activity against both Gram-negative and Gram-positive ones. A better effect against Gram-negative ones was displayed, contrary to the effect of the extract alone. This shift may be due to the antimicrobial activity that the solvent alone had towards the Gram-negative bacteria. Furthermore, none of the ingredients displayed activity against the yeast tested, *C. albicans*. Additionally, no ingredient displayed bactericidal activity. Regarding the MICs obtained, the extract with 1,2-hexanediol showed lower effective concentrations. 

Controls of the solvent (ingredients without extract) were also tested, and the results are listed in Table 2. It is possible to see that, in 1,2-pentanediol, the solvent control matches the activity of the ingredient in the case of *P. aeruginosa*, but not against *E. coli*, allowing us to conclude that the solvent alone does not match the antimicrobial activity of the ingredient. On the other hand, 1,2-hexanediol itself displays an MIC lower than the one obtained with the ingredient. 1,2-Hexanediol has been reported to be a moderately strong preservative in the cosmetic industry [18]. An MIC of between 1% and 2.5% has been reported for *S. aureus*, between 0.94% and 1.25% for *E. coli*, between 0.63% and 1% for *P. aeruginosa*, and between 0.94% and 1.25% for *C. albicans* [18,19,26]. As most of these reports used the 1,2-hexanediol at 100%, this may explain the differences verified between our results and those in the literature since, in this work, 1,2-hexanediol diluted in water at 25% (*v*/*v*) was used. Regarding 1,2-pentanediol, this solvent has been reported to exhibit antimicrobial activity against *S. aureus* at 10% [27]. Differences in antimicrobial activity observed between the extract, the extract mixed with the solvent, and the solvent alone may be attributed to the solvent’s varying affinity for specific phenolic groups, which are crucial in determining antimicrobial efficacy [16]. 

Overall, these results indicate that 1,2-hexanediol not only solubilizes the extract, but also acts as a booster in antimicrobial activity, since an increase in antimicrobial activity is observed when comparing the extract alone and the extract dissolved in 1,2-hexanediol. As this ingredient displays better antimicrobial activity, it was selected to perform the USP 51 challenge test.

### 2.3. Antimicrobial Effectiveness by USP 51 Challenge Test

The ingredient obtained by mixing the extract with 1,2-hexanediol at 5% (*v*/*v*) was added to O/W and W/O emulsions to evaluate how the ingredient behaves as a preservative once mixed with a wide composition of ingredients. Emulsions are the most complex cosmetic formulations, containing an aqueous phase and an oil phase that allows for the incorporation of a wide variety of active ingredients, such as emollients, thickeners, stabilizers, and emulsifiers. Also, O/W and W/O emulsion formulations can be tailored to provide different textures, where O/W are more fluid and light formulations, while W/O formulations are more compact, rich formulations, which are more susceptible to contamination. Therefore, O/W and W/O emulsions are excellent models due to their versatility and various ingredient compositions [28]. After adding the ingredient to the emulsion, a slight change in color (white to light brown) was observed, although no changes were observed in other the organoleptic properties, such as appearance, odor, and texture. The concentration used in this assay (5%) was chosen because it exhibited inhibitory activity against a broader range of microorganisms, effectively inhibiting more strains than at lower concentrations.

The microbial growth was controlled for 28 days, and the results are presented in Table 3. It was possible to conclude that, according to the USP 51 guidelines, the ingredient passed the challenge test at 5% (*v*/*v*) in both formulations for all the microorganisms tested. In the case of bacteria, a ≥ 2 log reduction was observed, and no increase was registered for the yeast and mold. Regarding the solvent control, this did not pass in the W/O formulation since a reduction of ≥2 log was not detected in *E. coli*. No growth was detected for *S. aureus* since the initial point, which may be due to the lack of nutrients in the O/W formulation leading to microbial death.

Previous works had studied the development of natural preservatives through the challenge test. An extract made from *Silene vulgaris* passed the challenge test between 10% and 20% (*w*/*w*) [1]. A *Santolina chamaecyparissus* extract passed the challenge test at 2% (*w*/*v*) [6]. A *Rubus rosaefolius* extract at 0.2% (*w*/*w*) concentration satisfied the criteria for microbial effectiveness [7]. The benchmark preservative commonly used in cosmetics is phenoxyethanol and has been reported to pass the challenge test at 0.5% (*w*/*w*) [5]. After evaluating the ingredient’s preservative potential, it was possible to conclude that a 5% (*v*/*v*) concentration is effective. 

The search for more natural products among consumers is putting pressure on manufacturers to respond to this need. Thus, reliance on new natural ingredients has been increasing. However, efficacy in preventing microbial contamination, avoiding the onset of unfavorable skin reactions, and their overall safety should be assured. Further analysis of the ingredients studied in this work, namely toxicity and irritability, should be performed in future work.

### 2.4. Ingredient Stability

To evaluate the capacity of the ingredient to keep their properties during its shelf life, a stability study was performed. The accelerated storage conditions were performed across 30 weeks, where 10, 20, and 30 weeks represent 1, 2, and 3 years of storage in real-time stability conditions, respectively. Phenolic compound and organic acid compositions, color, pH, and antioxidant capacity were evaluated during this time.

**Figure 1:**
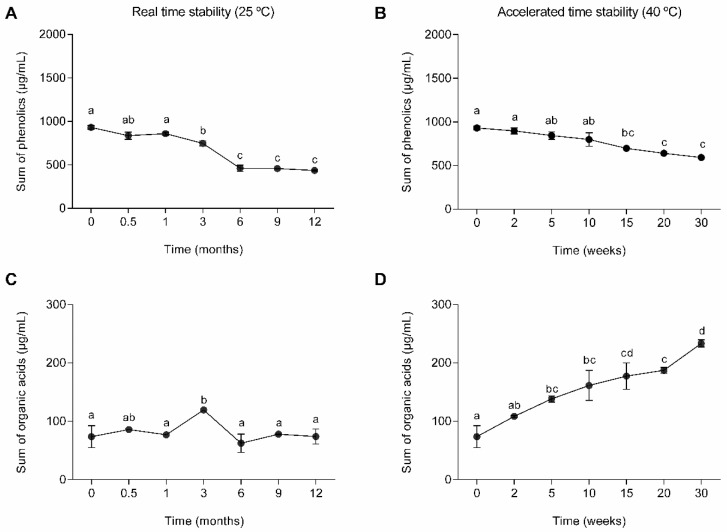
Sum of phenolics and organic acids (mean ± SD) (*n* = 3) in real-time (25 °C, 65% RH) and accelerated (40 °C, 65% RH) stability conditions. Different letters indicate statistical differences (*p* < 0.05) along storage. (**A**) Sum of phenolics along 12 days in real-time (25 °C, 65% RH) stability conditions. (**B**) Sum of phenolics along 12 days in accelerated (40 °C, 65% RH) stability conditions. (**C**) Sum of organic acids along 12 days in real-time (25 °C, 65% RH) stability conditions. (**D**) Sum of organic acids along 12 days in accelerated (40 °C, 65% RH) stability conditions.

**Figure 2:**
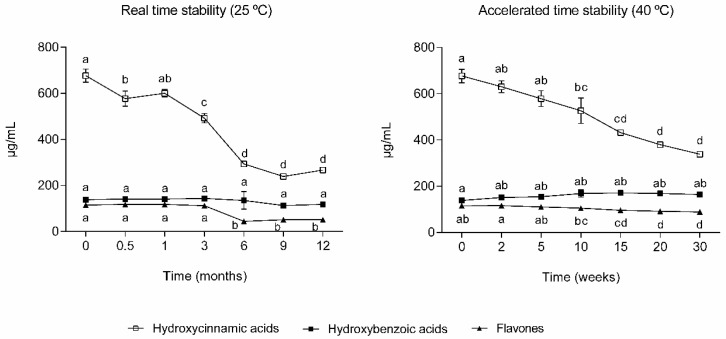
Phenolic compounds composition of the ingredient (mean ± SD) (*n* = 3) during real-time (25 °C, 65% RH) and accelerated (40 °C, 65% RH) stability conditions. Different letters indicate statistical differences (*p* < 0.05) along storage.

The phenolic compounds and organic acid concentrations throughout the ingredient stability testing were analyzed through LC–MSqTOF, and the results are represented in Figure 1. Furthermore, the phenolic profile was also evaluated, and the main classes are presented in Figure 2. In real-time stability conditions, it was possible to see those phenolic compound concentrations decrease (*p* < 0.05) during the storage time (Figure 1A), while the organic acids (quinic acid, malic acid, citric acid, sebacic acid, azelaic acid and aconitic acid) remained stable over time (*p* > 0.05) (Figure 1C). In the hydroxybenzoic acid class, the most representative compounds were 1-O-vanilloyl-beta-D-glucose (↓13%), vanillic acid (↑15%), 2,5-dihydroxybenzoic acid (↓65%), 4-hydroxybenzoic acid (↓5%), and 3,4-hydroxybenzaldehyde (↑35%), presenting different behaviors throughout the storage. In the hydroxycinnamic acid class, the most representative compounds were neochlorogenic acid (↓65%), chlorogenic acid (↑57%), 4-caffeoylquinic acid (↓78%), and trans-3-feruloylquinic acid (↓35%), presenting mainly a decrease during the study. Lastly, in the less representative class were the flavones, where the most representative compounds were luteolin-8-C-glucoside (↓55%), apigenin 7-*O*-neohesperidoside (↑52%), and 3′,5′-*O*-dimethyltricetin (↓35%).Hydroxybenzoic acids were the ones with higher stability, not presenting significant changes (*p* > 0.05) over the 12 months (Figure 2), resulting from a balance between some compounds which increased and others that decreased. On the other hand, the flavones and hydroxycinnamic acids decreased (*p* < 0.05) between 55% and 61%. These findings are supported by the literature. Previous works have reported the decrease in phenolic compounds during storage at room temperature [29,30]. Furthermore, flavones and hydroxybenzoic acids were reported to be the most stable compounds, while hydroxycinnamic acids were the less stable ones [29]. Phenolic compounds’ stability varies significantly as some are relatively stable and others are volatile, thermolabile, and prone to oxidation [31]. Hydroxycinnamic acids are prone to degradation and can generate other compounds, such as hydroxybenzoic acids [32]. 

In accelerated stability conditions, the sum of all quantified phenolic compounds decreased by 36% after 30 weeks (Figure 1B), which corresponds to 3 years in real-time stability conditions, while after 10 weeks, the decrease was 14%, which corresponds to 1 year. Organic acid (quinic acid, malic acid, citric acid, sebacic acid, azelaic acid and aconitic acid) content increased (*p* < 0.05) throughout the storage (Figure 1D), which can indicate a possible microbial contamination of the formulation. Among the organic acids that increased was malic acid, and this compound has been reported to increase at 40 °C [33], caused by the activation of its metabolic formation during the glycolytic pathway as a product of the transformation of succinic acid [34]. In turn, succinic acid could have been produced by bacterial metabolism in a process known as cross feeding, a phenomenon in which microorganisms exchange metabolites or nutrients, facilitating their growth and survival [35].

The hydroxycinnamic acids are the most representative class of polyphenols present in the ingredient and were the ones with higher depletion (*p* < 0.05) throughout the storage period (Figure 2) with a decrease of 22% after 10 weeks (1-year real-time conditions) and of 50% after 30 weeks (3 years real-time conditions) in storage. The flavones also significantly decreased (*p* < 0.05), presenting a decrease of 9% after 10 weeks (1-year real-time conditions) and of 23% after 30 weeks (3 years real-time condition). Contrarily, the hydroxybenzoic acids increased over storage, with a maximum variation of 22%. Among these are 4-hydroxybenzoic acid, 3,4-hydroxybenzaldehyde, and protocatechuic acid, the increase in which were probably derived from *p*-hydroxybenzoic acid [36,37].

**Figure 3:**
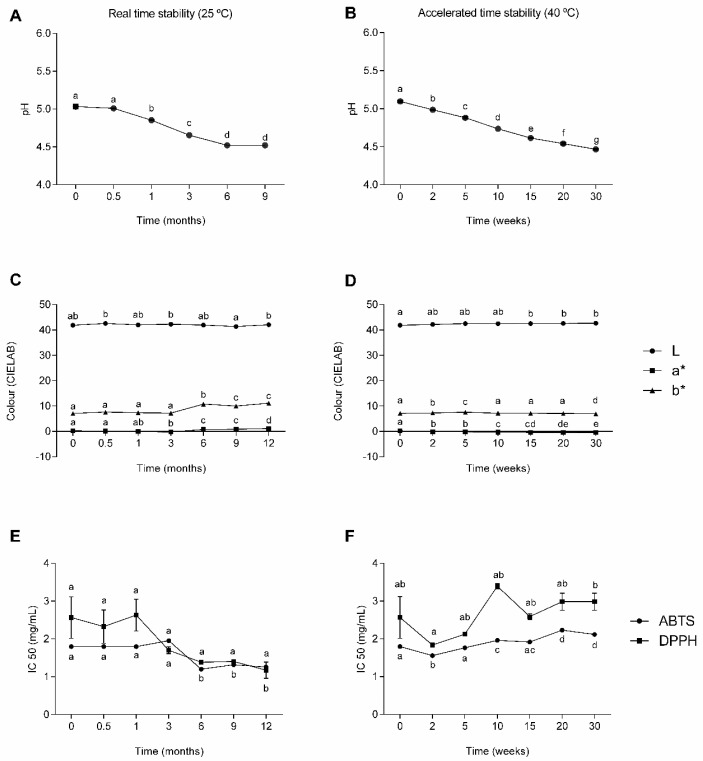
Results of pH value, color, and antioxidant capacity (ABTS and DPPH) of the ingredient (mean ± SD) (*n* = 3) during real-time (25 °C, 65% RH) and accelerated (40 °C, 65% RH) stability conditions. Different letters indicate statistical differences (*p* < 0.05) along storage. pH values during real-time (**A**) and accelerated (**B**) stability conditions, CIELAB colour during real-time (**C**) and accelerated (**D**) stability conditions, antioxidant activity during real-time (**E**) and accelerated (**F**) stability conditions.

The ingredient’s pH was monitored, and a decrease was observed. In real-time stability conditions, a decrease of 11% occurred, from 5.10 to 4.52 after 12 months, although the first month was the period that showed a significant decrease (*p* < 0.05) (Figure 3A). Under accelerated stability conditions, the pH decrease was significant (*p* < 0.05) over the storage period, with a decrease of 12%, from 5.10 to 4.47 after 30 weeks (Figure 3B). This acidification could be related to the dissociation of the organic acids [38]. Nevertheless, the ingredient‘s pH remained acidic, between 4.5 and 5.5, which is the skin’s natural pH range. This characteristic is extremely important in regulating protective functions (e.g., preventing colonization of pathogenic bacteria), maintaining the lipid barrier homeostasis, and the integrity of the stratum corneum [39,40]. Furthermore, acidification during storage has been reported in cosmetics formulated with phenolic compound extracts [41]. Therefore, although significant differences in sugarcane-based ingredient pH values were observed throughout time, these differences do not affect the overall acidity of the ingredient.

To measure any representative color change, the CIELAB parameters (L, a* and b*) were evaluated. In real-time stability conditions, the color intensity decreased (*p* < 0.05) after 1 month (Figure 3C), while in the accelerated stability conditions, the changes started after the 2-week time point (Figure 3D). These differences were not detectable by the naked eye. Color changes during the storage period can be explained by the presence of hydroxycinnamic acids in the ingredient, since these have been reported to be involved in oxidative browning reactions that can contribute to changes in color [42].

As an extra indicator of the preservative potential that improved oxidative stability and bioactive activity, the antioxidant activity was measured throughout storage, since the sugarcane straw extract was previously reported to have an antioxidant capacity [8]. The antioxidant capacity was measured according to two chemical methods (ABTS and DPPH), and the IC_50_ was calculated. In real-time stability conditions, there was a visible decrease of 30% in the IC_50_ for the ABTS and 54% in DPPH method (Figure 3E), indicating that it was necessary to reduce the quantity of the extract to achieve a 50% inhibition of the free radicals, although the antioxidant capacity only changed significantly (*p* < 0.05) after 3 months. As opposed to what happened in the real-time stability conditions, in the accelerated stability conditions, there was a visible increase of 17% in the IC_50_ for both methods (Figure 3F), indicating that it was necessary to increase the quantity of the extract to achieve a 50% inhibition of the free radicals. Furthermore, the changes (*p* < 0.05) were visible immediately after the initial time point. The increase in the antioxidant capacity can be explained by the increase in the organic acid and hydroxybenzoic acid contents, such as protocatechuic acid and malic acid. These compounds are described as having scavenging properties [43,44]. Previous studies have also reported, in both real-time and accelerated stability conditions, a decrease in the antioxidant capacity of natural extracts composed of phenolic compounds [45,46]. 

Several factors may influence the stability of preservatives such as the solubility in the O/W or W/O emulsions, pH, and temperature during use [21]. After the stability assay, it was possible to conclude that a one-month storage at room temperature would be ideal for higher ingredient stability, which could be extended if stored under refrigerated conditions. These results may be challenging, since most cosmetic products are indicated to have a shelf life of between 2 and 3 years [47]. Based on the results of our ingredients after 3 years, the phenolic compounds reduced by 36%, while the organic acids increased by 217%, and the antioxidant activity increased by 17%, indicating that the preservative activity of the ingredient may be conserved. 

Nevertheless, strategies to improve the ingredient shelf-life stability should be considered. The next step would be to evaluate the stability of the ingredient within a complete formulation, as other cosmetic ingredients may contribute to the synergistic and booster effects of the preservative. Should stability issues persist, additional booster ingredients, such as Vitamin E and essential oils, could be added to enhance preservation [5]. While refrigerated conditions might improve stability, this approach is less favorable due to the challenges it poses for commercialization and consumer use. 

## 3. Materials and Methods

### 3.1. Extraction and Development of the Cosmetic Ingredient

Extraction and purification of phenolic compounds from sugarcane straw was carried out as described by Carvalho et al. (2013) [8]. Briefly, the straw was provided by Raízen from São Paulo, Brazil and, upon arrival, it underwent a milling and drying process. Firstly, the straw was dried at 40 °C over 16 h using a ventilated oven (Memmert GmbH + Co.KG, Schwabach, Germany). Afterwards, a milling process was performed utilizing a grinder (SM100, Retsch, Hann, Germany) to obtain a particle size of smaller than 4 mm. The straw was then stored at room temperature in the dark.

The extraction protocol was performed over 24 h with a 50% ethanol/water solution, with a ratio biomass/solvent of 1:10 (*w*:*v*). The obtained extract was purified using amberlite XAD-2 (Sigma-Aldrich, St. Louis, MO, USA) in a 1:2 (*w*/*v*) ratio while being shaken at 100 rpm overnight. The resin was then collected and thoroughly cleaned with deionized water at pH 2 to remove any sugar that had been adsorbed. A solution of 50% ethanol (*v*:*v*), pH 2 (HCl, 10 M), was used to desorb the phenolic compounds overnight at 100 rpm and 37 °C. Decantation and filtering (type I filter, VReis, Lisbon, Portugal) were used to recover the ethanolic extract. The ethanol was then evaporated through rotary evaporation (40 °C, 150 mbar) (Heidolph, Walpersdorfer, Germany), and the extract was freeze-dried (Martin Christ, Osterode am Harz, Germany) thus obtaining a powder. 

For the development of a cosmetic ingredient based in sugarcane straw extract, the following 9 cosmetic solvents were tested: 1,3-propanediol, 1,2-hexanediol, dipropylene glycol, 1,3-butanediol, 1,2-pentanediol, 1,5-pentanediol, caprylyl glycol (Sigma-Aldrich, St. Louis, MO, USA), glycerin (Acofarma, Madrid, Spain) and ethylene glycol (Acros Organics, Geel, Belgium). Different proportions of these solvents were tested, namely 75:25%, 50:50%, 25:75% in water. 

### 3.2. Antimicrobial Activity

#### 3.2.1. Microorganisms

The microorganisms studied were *Staphylococcus aureus* (DSM 799), *Pseudomonas aeruginosa* (DSM 1128), *Escherichia coli* (DSM 1576), *Candida albicans* (DSM 1386), and *Aspergillus brasiliensis* (DSM 1988), all of which were acquired by the Leibniz Institute DSMZ.

#### 3.2.2. Broth Microdilution Assay

Antimicrobial activity was evaluated by the determination of the minimum inhibitory concentration (MIC_80_) and the minimum bactericidal concentration (MBC) of the extract and the engineered ingredients by employing a broth microdilution assay, according to the standards for aerobic bacteria [48] and yeasts [49]. 

Briefly, a 5% (*w*/*v*) stock solution of the extract and each ingredient were prepared in the required medium (Mueller–Hinton broth for bacteria and Roswell Park Memorial Institute (RPMI) 1640 for yeasts) and filtered with a 0.22 μm filter (Frilabo, Maia, Portugal), where the solutions were diluted in the medium at 4, 3, 2, 1 and 0.5% (*w*/*v*) and then tested. The microorganisms were inoculated at 2% (*v*/*v*) from an inoculum standardized with an 0.5 McFarland standard (ca. 10^8^ CFU/mL) and then adjusted to obtain a final inoculum concentration of 10^5^ CFU/mL in the testing solutions. The assay was performed in a 96-well microplate (Sarstedt, Nümbrecht, Germany) for 24 h at 37 °C when testing for aerobic bacteria, and an incubation period of 48 h at 30 °C was applied when testing for yeasts. The optical density (OD) was measured using a microplate reader (Epoch, Biotek, Winooski, Vermont, EUA) each hour at 625 nm. All measurements were performed in duplicate, and two independent assays were completed. Afterwards, the concentrations for which no growth was detected were plated to validate the MBC.

#### 3.2.3. Challenge Test Protocol

The challenge test, according to the USP 51 standard [50], was performed to assess the preservative capacity of the ingredient in a cosmetic formulation. For that purpose, the ingredient was incorporated in two different formulations—water–oil (W/O) and oil–water (O/W) formulations—as described in Table 4 and Table 5. To prepare the W/O formulation, first, phases I and II were heated at 70 °C and then phase II was slowly added to phase I with high-speed mixing. After cooling, the preservative ingredient was added, and the formulation was mixed until it was homogeneous. To prepare the O/W formulation, phases I and III were heated to 70 °C. Then, phase II was pre-mixed and added to phase I with mixing. Phases IV and II were mixed and added to phase I–II with high-speed mixing for 5–7 min. The formulation was cooled with slow mixing and the preservative ingredient was added. 

The cosmetic formulations were inoculated at 1% (*v*/*w*) from an inoculum standardized with an 0.5 McFarland standard (ca. 10^8^ CFU/mL) and then adjusted to obtain a final inoculum concentration of 10^5^ CFU/mL in the emulsions. The microorganisms, *S. aureus*, *P. aeruginosa*, *E. coli*, *C. albicans* and *A. brasiliensis*, were inoculated and incubated at room temperature for 28 days. At each time point (0, 7, 14, and 28 days), serial dilutions were performed and plated on tryptic soy agar (TSA) (Biokar Diagnostics, Allonne, France) and incubated at 30 °C for 24 h (bacteria) or plated on sabouraud dextrose agar (SDA) (Biokar Diagnostics, Allonne, France) and incubated at 25 °C for 48 h (yeasts and molds). After the 28-day assay, the ingredients were evaluated for their effectiveness according to the USP 51 standard guidelines for category 2 products (topically used products made with aqueous bases or vehicles, nonsterile nasal products, and emulsions, including those applied to mucous membranes) (Table 6). All assays were performed in duplicate. Results were expressed as follows:

∆Log CFU/mL=Initial Log CFU/mL−Final Log CFU/mL

### 3.3. Ingredient Stability Tests

The best performing ingredient (20% dry extract dissolved in 25% of 1,2-hexanediol and 75% of water) was kept in glass amber containers and stored at 25 °C and 60% relative humidity (RH) (real-time stability conditions) for 12 months as well as 40 °C and 75% RH (accelerated stability conditions) for 30 weeks, following the ISO/TR18811 “Cosmetics—Guidelines on the stability testing of cosmetic products” [51]. At each time point (Table 7), the color, pH, phenolic content, and antioxidant capacity were measured.

#### 3.3.1. Phenolic Compounds and Organic Acid Identification and Quantification

Phenolic compound and organic acid identification and quantification were carried out by LC-ESI-UHR-QqTOF-MS as described by Oliveira et al. (2105) [52]. 

For this analysis, the ingredient was prepared at 50 mg/mL and was filtered in a 0.45 µm syringe filter (Macherey-Nagel, Dueren, Germany). After injecting 5 µL of samples, the separation was performed in a UHPLC from the Bruker Elute series, coupled with an ultrahigh resolution Qq-time-of-flight (UHR-QqTOF) mass spectrometer with 50,000 full-sensitivity resolution (FSR) (Impact II, Bruker Daltonics, Bremen, Germany). Separation of metabolites was performed using a BRHSC18022100 intensity Solo 2 C18 (100 × 2.1 mm, 2.2 μm, Bruker) carried out over 26 min (Figure S1) under the following gradient conditions: 0 min, 0% B; 10 min, 21.0% B; 14 min, 27% B; 18.30 min, 58%; 20.0 min, 100%; 24.0 min, 100%; 24.10 min, 0%; 26.0 min, 0% at a flow rate of 0.25 mL/min. Mobile phases were 0.1% (*v*/*v*) aqueous formic acid (solvent A) and acetonitrile with 0.1% (*v*/*v*) formic acid (solvent B). Negative ionization mode was used to set the parameters for the MS analysis, and spectra from *m*/*z* 20 to 1000 were collected using the Auto MS scan mode. Compounds were identified based on the precise mass [M–H] of each one, as seen in Table S1. With the help of calibration curves for standards (Sigma-Aldrich, St. Louis, MO, USA), quantification was accomplished. The results were expressed in µg/g dw of the extract.

#### 3.3.2. pH and Color Measurement

The samples’ pH was measured with a SevenCompact pH meter (Mettler Toledo, Urdorf, Switzerland).

To measure any representative color change in the ingredient during storage, a 10× dilution in water was made, and the color was measured with a colorimeter (Konica Minolta CR-400 chromameter) equipped with a D65 illuminant and the observer at 2°. The instrument was calibrated using a white reference tile (Y 93.9; x 0.3159, y 0.3322). Color was expressed according to the CIELAB space, defined by the International Commission on Illumination and expressed as the following three values: L* for the lightness from black (0) to white (100), a* from green (−) to red (+), and b* from blue (−) to yellow (+). 

#### 3.3.3. Antioxidant Capacity Evaluation

##### ABTS Radical Cation Decolorization Assay

The 2,2′-azino-bis (3-ethylbenzothiazoline-6-sulphonic acid) (ABTS) decolorization assay was performed as described by Gonçalves et al. (2009) [53]. A solution of ABTS was attained by adding a 7 mmol/L solution of ABTS salt (Sigma, St. Louis, MO, USA) to 2.45 mM of K_2_S_2_O_8_ (Merck, Darmstadt, Germany) at a 1:1 (*v*/*v*) proportion. The solution was left in the dark for 16 h and then diluted with deionized water to obtain an OD of 0.700 ± 0.020 at 734 nm. 

In a microplate reader (Synergy H1 microplate reader, Biotek, Winooski, Vermont, EUA), samples were prepared at five different concentrations (1:1 dilution starting at a concentration of 3.123 mg/mL), and 15 µL of each sample was placed in a microplate with 200 µL of ABTS. The incubation period was five minutes at 30 °C. After that, the OD was measured at 734 nm. Trolox standard solutions (0.075–0.008 mg/mL) (Sigma-Aldrich, St. Louis, MO, USA) were used to create the calibration curve. The assays were carried out in duplicate. Half the maximal inhibitory concentration (IC_50_) (mg/g dw) was used to express the results.

##### DPPH Radical Cation Decolorization Assay

The 2,2-diphenyl-1-picrylhydrazyl (DPPH) decolorization assay was performed as described by Gonçalves et al. (2009) [53]. A DPPH solution (Sigma-Aldrich, St. Louis, MO, USA) was prepared at 600 μM using methanol and diluted to obtain an OD of 0.600 ± 0.100 at 515 nm. Samples were prepared at five different concentrations (1:1 dilution, starting from a 3.123 mg/mL concentration), and 25 μL of each sample were placed in a microplate with 175 μL of DPPH solution. The plate was incubated at room temperature for 30 min, following the measurement of the OD at 515 nm in a microplate reader. The calibration curve was prepared using the Trolox standard solutions (0.075–0.008 mg/mL). Assays were performed in duplicate. The results were expressed in IC_50_ (mg/g dw).

### 3.4. Statistical Analysis

IBM SPSS statistics (v. 21, 2012) (New York, NY, USA) software was used for statistical analysis. The Shapiro–Wilk test was used to see if the data showed a normal distribution. When a normal distribution was found, a one-way ANOVA test was conducted to evaluate whether the differences were statistically significant at a 5% confidence level.

## 4. Conclusions

The cosmetic industry is seeking new natural and sustainable preservatives for their formulations due to their expected lower toxicity and side effects. For this goal, a cosmetic ingredient was formulated based on a sugarcane straw extract, which is rich in phenolic compounds. Several cosmetic solvents were tested as solubilizing agents, and 1,2-hexanediol displayed better solubility and boosted antimicrobial performance. This developed ingredient displayed an MIC of between 3% and 5% (*w*/*v*) against *S. aureus*, *E. coli* and *P. aeruginosa*. Additionally, the ingredient also obeyed the criteria of the USP 51 challenge test at 5% (*w*/*v*) in the W/O and O/W emulsions.

The ingredient was also subject to a real-time and accelerated stability test, by which was possible to conclude that, at room temperature, ingredient stability could be maintained over a one-month storage period, although this period could be extended under refrigerated conditions. 

Overall, the developed preservative ingredient shows potential as a natural preservative ingredient for the cosmetic industry.

## Figures and Tables

**Table 1:** Ingredients formulated with sugarcane straw extract and cosmetic solvents. Soluble states the extract was completely dissolved in the mixture.

Extract (%)	Solvent	Proportion Solvent/Water (%, *v*/*v*)	Soluble
≤20	1,3-Propanediol	75:25	No
		50:50	No
		25:75	No
≤20	1,2-Hexanediol	75:25	No
		50:50	No
		25:75	Yes
≤30	Dipropylene Glycol	75:25	No
		50:50	Yes
		25:75	No
≤20	1,3-Butanediol	75:25	No
		50:50	No
		25:75	No
≤20	1,2-Pentanediol	75:25	No
		50:50	No
		25:75	Yes
≤20	1,5-Pentanediol	75:25	No
		50:50	No
		25:75	Yes
≤20	Caprylyl Glycol	75:25	No
		50:50	No
		25:75	No
≤20	Glycerin	75:25	No
		50:50	No
		25:75	No
≤20	Ethylene Glycol	75:25	No
		50:50	No
		25:75	No

**Table 2:** MICs of the sugarcane straw extract and the different ingredients tested against common cosmetic pathogen microorganism (*n* = 2). The percentage of extract is in *w*/*v* and solvents is expressed in *v*/*v*.

Ingredient	Microorganism	MIC (%)
Straw extract (without solvent)	*S. aureus*	5
	*E. coli*	5
	*P. aeruginosa*	-
	*C. albicans*	-
30% straw extract + 25% 1,2-pentanediol + 75% H_2_O	*S. aureus*	-
	*E. coli*	5
	*P. aeruginosa*	5
	*C. albicans*	-
25% 1,2-pentanediol + 75% H_2_O (solvent control)	*S. aureus*	-
	*E. coli*	-
	*P. aeruginosa*	5
	*C. albicans*	-
20% straw extract + 25% 1,2 hexanediol + 75% H_2_O	*S. aureus*	5
	*E. coli*	3
	*P. aeruginosa*	4
	*C. albicans*	-
25% 1,2 hexanediol + 75% H_2_O (solvent control)	*S. aureus*	5
	*E. coli*	2
	*P. aeruginosa*	3
	*C. albicans*	5

**Table 3:** Log (CFU/mL) variation of the cosmetic contaminants after 28 days of storage, according USP51 challenge test (*n* = 3).

Formulation	∆ Log CFU/mL				
	*S. aureus*	*E. coli*	*P. aeruginosa*	*C. albicans*	*A. brasiliensis*
Ingredient O/W	No growth	−5.66	−5.2	−4.77	−5.9
Solvent control O/W	No growth	−4.8	−5.13	−4.83	−0.91
Ingredient W/O	−5.03	−4.4	−4.61	−3.6	−1.95
Solvent control W/O	−5.29	−1.88	−5.08	−3.61	−1.14

**Table 4:** Composition of the formulation recipe of water/oil emulsion.

Vendor	Ingredient	% (*w*/*v*)
Phase I		
AAk	Akoline PGPR	5.00
Aprinnova	Neossance Squalene	5.00
Acofarma	Caprylic/Capric triglyceride	7.00
	Vaseline	10.00
	Lanoline	10.00
	Beeswax	1.80
	Magnesium Stearate	1.00
Phase II		
Local Source	Deionized water	51.45
Acofarma	Glycerin	3.00
	Sodium Chloride	0.75
Phase III		
	Preservative	5.00
Total		100.00

**Table 5:** Composition of the formulation recipe of oil/water emulsion.

Vendor	Ingredient	% (*w*/*v*)
Phase I		
Local Source	Deionized Water	43.35
Acofarma	Organic Aloe Vera 1x	20.00
	Phase II	
Acofarma	Glycerin	5.00
	Xanthan Gum	0.15
Phase III		
Acofarma	Montanov 68	3.00
	Lanette OOR	1.00
	Caprylic/Capric Triglyceride	15.00
	Lanolina	1.00
Aprinnova	Neossance Squalane	5.00
Phase IV		
Seppic	Sepinov EMT	0.50
	SIMULGEL^TM^ EPG	1.00
Phase V		
	Preservative	5.00
Phase VI		
Sigma	Sodium Hydroxide 50% (pH 5.5–6.0)	qs 100
Total		100.00

**Table 6:** USP 51 challenge test criteria for category 2 products.

Bacteria	Not less than 2.0 log reduction from the initial count at 14 days, and no increase from the 14 days count at 28 days.
Yeast and Molds	No increase (not more than 0.5 log10 unit higher than the previous value measured) from the initial calculated count at 14 and 28 days.

**Table 7:** Ingredient stability conditions and time points.

Condition	Time Point						
25 °C, 60% RH	0	0.5 months	1 month	3 months	6 months	9 months	12 months
40 °C, 75% RH	0	5 weeks	10 weeks ^1^	15 weeks	20 weeks ^2^	30 weeks ^3^	

^1^ Represents stability for 12 months at real time; ^2^ Represents stability for 24 months at real time; ^3^ Represents stability for 36 months at real time.

## Data Availability

Data will be made available upon request.

## Supplementary Materials

The following supporting information can be downloaded at: https://www.mdpi.com/article/10.3390/molecules29163928/s1.
