# Supplementary material for: New Natural and Sustainable Cosmetic Preservative Based on Sugarcane Straw Extract
Source: Molecules. 2024 Aug 20;29(16):3928. doi: 10.3390/molecules29163928 (PMC11356945; doi:10.3390/molecules29163928)

Table S1. LC-ESI-UHR-QqTOF-MS data of organic acids and phenolic compounds identified in ethanolic extracts obtained from sugarcane straw.

Name	Formula-H	m/z Measured [M-H]-	MS/MS fragments (m/z)
Organic acids			
Quinic acid	$C_7H_{11}O_6$	191.0	85, 75, 59
Malic acid	$C_4H_6O_5$	133.0	71, 115, 59
Citric acid	$C_6H_7O_7$	191.1	111, 87, 57,67
Sebacic acid	$C_{10}H_{17}O_4$	201.1	139, 183, 201
Azelaic acid	$C_9H_{16}O_4$	187.1	125, 169, 187
Aconitic acid	$C_6H_6O_6$	173.0	
Hydroxybenzoic acids			
1-O-Vanilloyl-β-D-glucose	$C_{14}H_{17}O_9$	329.0	167
Vanillic acid	$C_8H_7O_4$	167.0	108, 119, 152
Protocatechuic acid	$C_7H_5O_4$	153.0	109, 153
2,5-Dihydrobenzoic acid	$C_7H_5O_4$	153.0	109, 153
2,4 Dihydrobenzoic acid	$C_7H_5O_4$	153.0	109, 153
Gentisic acid 2-*O*-β-glucoside	$C_{13}H_{15}O_9$	315.1	108, 152
Gentisic acid 5-*O*-β-glucoside	$C_{13}H_{15}O_9$	315.1	109, 153
Protocatechuic acid 4-β-glucoside	$C_{13}H_{15}O_9$	315.1	109, 153
4-Hydroxybenzoic acid	$C_7H_5O_3$	137.0	137
3,4-Dihydroxybenzaldehyde	$C_7H_5O_3$	137.0	93, 137
4-Hydroxybenzaldehyde	$C_7H_5O_2$	121.0	121
Hydroxybenzoic-4- β-glucoside	$C_{13}H_{15}O_8$	299.1	108, 152
Hydroxycinnamic acids			
Neochlorogenic acid	$C_{16}H_{17}O_9$	353.1	135, 179, 191
Chlorogenic acid	$C_{16}H_{17}O_9$	353.1	191
4-Caffeoylquinic acid isomer 1	$C_{16}H_{17}O_9$	353.1	135, 173, 179, 191
4-Caffeoylquinic acid isomer 2	$C_{16}H_{17}O_9$	353.1	191
cis-5-*O*-p-Coumaroylquinic acid isomer 1	$C_{16}H_{17}O_8$	337.1	119, 163
cis-5-*O*-p-Coumaroylquinic acid isomer 2	$C_{16}H_{17}O_8$	337.1	93, 163, 173, 191
cis-5-*O*-p-Coumaroylquinic acid isomer 3	$C_{16}H_{17}O_8$	337.1	191
3-*O*-Feruloylquinic acid	$C_{17}H_{19}O_9$	367.1	134, 193
trans-3-Feruloylquinic acid	$C_{17}H_{19}O_9$	367.1	173
Caffeic acid	$C_9H_8O_4$	179.0	135, 179
Ferulic acid	$C_{10}H_9O_4$	193.1	134
Isoferulic acid	$C_{10}H_9O_4$	193.0	134, 161, 193
p-Coumaric acid	$C_9H_7O_3$	163.0	119
p-Coumaric acid isomer	$C_9H_7O_3$	163.0	119
Caffeoylquinic acid	$C_{16}H_{18}O_9$	515.1	515
1,3-Dicaffeoylquinic acid	$C_{25}H_{23}O_{12}$	515.1	173, 179, 191, 335, 353
Caffeoylshikimic acid	$C_{25}H_{23}O_{14}$	335.1	135, 161, 179
1,3-Dicaffeoylquinic acid	$C_{25}H_{24}O_{12}$	335.1	135, 161, 179
Flavones			
Apigenin-8-C-glucoside isomer 1	$C_{21}H_{19}O_{10}$	431.1	89, 179

Apigenin-8-C-glucoside isomer 2	$C_{21}H_{19}O_{10}$	431.1	311, 341, 431
Apigenin-6-C-glucoside	$C_{21}H_{19}O_{10}$	431.1	311, 341
Isoschaftoside	$C_{26}H_{27}O_{14}$	563.1	353, 473
Neoschaftoside	$C_{26}H_{27}O_{14}$	563.1	399, 473
Apigenin-6-C-arabinoside-8-C-glucoside	$C_{26}H_{27}O_{14}$	563.1	293, 413
Luteolin-8-C-glucoside	$C_{21}H_{19}O_{11}$	447.1	327, 357
Vitexin 2''-*O*-beta-L-rhamnoside	$C_{27}H_{29}O_{14}$	577.2	293, 413
Apigenin 7-*O*-neohesperidoside isomer 1	$C_{27}H_{29}O_{14}$	577.2	293, 413, 473
Apigenin 7-*O*-neohesperidoside isomer 2	$C_{27}H_{29}O_{14}$	577.2	293, 413, 474
6-Methoxyluteolin 7-rhamnoside	$C_{22}H_{21}O_{11}$	461.1	461
Tricin-*O*-neohesperoside isomer 1	$C_{29}H_{33}O_{16}$	637.2	329
Tricin-*O*-neohesperoside isomer 2	$C_{29}H_{33}O_{16}$	637.2	329
Tricin-7-*O*-glucoside	$C_{25}H_{31}O_{10}$	491.2	329
Tricin-7-*O*-rhamnosyl-glucuronide	$C_{29}H_{31}O_{17}$	651.2	329
Tricin-4-(*O*-erythro) ether glucoside isomer 1	$C_{33}H_{35}O_{16}$	687.2	165, 195, 329, 491, 525
Tricin-4-(*O*-erythro) ether glucoside isomer 2	$C_{33}H_{35}O_{16}$	687.2	165, 195, 329, 491, 526
Tricin	$C_{17}H_{13}O_{7}$	329.1	299

Figure S1. LC-ESI-UHR-QqTOF-MS chromatogram of organic acids and phenolic compounds identified in the sugarcane straw extract-based ingredient.

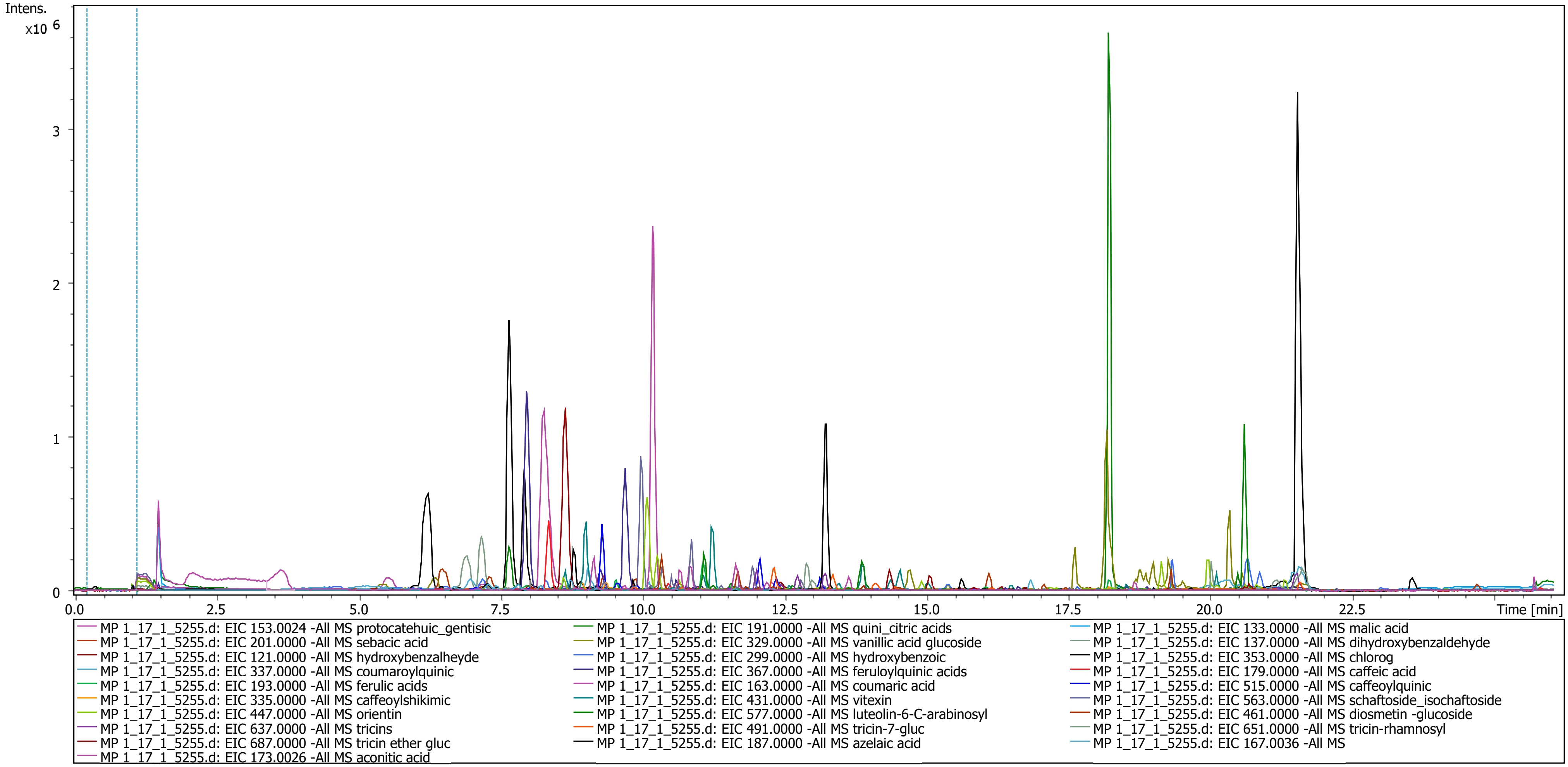

Supplement: Supplementary file 1 [file molecules-29-03928-s001.zip › molecules-3099745-supplementary.pdf]
